# Composite RGO/Ag/Nanosponge Materials for the Photodegradation of Emerging Pollutants from Wastewaters

**DOI:** 10.3390/ma17102319

**Published:** 2024-05-14

**Authors:** Ettore Madonia, Antonella Di Vincenzo, Alberto Pettignano, Roberto Scaffaro, Emmanuel Fortunato Gulino, Pellegrino Conte, Paolo Lo Meo

**Affiliations:** 1 Department of Food, Agriculture, Food and Forest Sciences, University of Palermo, V.le delle Scienze ed. 4, 90128 Palermo, Italy; ettore.madonia@unipa.it (E.M.); pellegrino.conte@unipa.it (P.C.); 2 Department of Biological, Chemical and Pharmaceutical Sciences and Technologies, University of Palermo, V.le delle Scienze ed. 17 “S. Cannizzaro”, 90128 Palermo, Italy; 3 Department of Physics and Chemistry “E. Segrè”, University of Palermo, V.le delle Scienze ed. 17 “S. Cannizzaro”, 90128 Palermo, Italy; alberto.pettignano@unipa.it; 4 Department of Engineering, University of Palermo, V.le delle Scienze ed. 6, 90128 Palermo, Italy; roberto.scaffaro@unipa.it (R.S.); emmanuelfortunato.gulino@unipa.it (E.F.G.)

**Keywords:** nanosponges, reduced graphene oxide, silver nanoparticles, photocatalysis, emerging pollutants

## Abstract

Some composite materials have been prepared, constituted by a cyclodextrin-*bis*-urethane-based nanosponge matrix in which a reduced graphene oxide/silver nanoparticles photocatalyst has been dispersed. Different chain extenders were employed for designing the nanosponge supports, in such a way as to decorate their hyper-cross-linked structure with diverse functionalities. Moreover, two different strategies were explored to accomplish the silver loading. The obtained systems were successfully tested as catalysts for the photodegradation of emerging pollutants such as model dyes and drugs. Enhancement of the photoactive species performance (up to nine times), due to the synergistic local concentration effect exerted by the nanosponge, could be assessed. Overall, the best performances were shown by polyamine-decorated materials, which were able to promote the degradation of some particularly resistant drugs. Some methodological issues pertaining to data collection are also addressed.

## 1. Introduction

Increasing concerns have risen in recent years, because of the massive presence of emerging pollutants in urban as well as industrial wastewater [1,2,3,4]. In particular, the presence in water bodies of drugs such as antibiotics or anti-inflammatories constitutes a serious threat because of the potential consequences on the development and diffusion of antibiotic-resistant bacterial strains [5,6]. This, in perspective, may lead to serious sanitary emergencies. Among the different strategies for wastewater treatment, specifically devoted to eliminating pollutants such as drugs or dyes, photo-oxidative degradation is undoubtedly one of the more explored and extensively investigated [6,7,8]. The general principles on which this approach relies are well assessed. In brief, generating ROS (Reactive Oxygen Species) upon irradiation of a semiconductor photoactive species may lead to effective oxidative degradation, up to complete mineralization, of an organic substrate [6,9,10]. As accounted for by a massive amount of literature, various photocatalysts have been investigated for this purpose. In particular, significant attention has been attracted by composite materials in which a noble metal (Au, Pt, Ag) or an oxide (TiO_2_, ZnO, etc.) in nanoparticle form [11,12,13,14,15,16,17,18,19,20] is associated with a carbonaceous material such as graphene (G), graphene oxide (GO), or reduced graphene oxide (RGO) [21,22,23,24,25,26]. 

We have recently shown that significant activity enhancement can be achieved in this context by associating the photoactive species with a functional sorbent material such as a nanosponge (NS) [27,28]. In brief, NSs [29,30,31] are hyper-reticulated polymers constituted by supramolecular host units—cyclodextrins (CDs) in the most common cases, but also calixarenes (CAs) occasionally [32,33]—held together by suitable linker units. These materials are able to adsorb up to a significant extent diverse suitably structured organic species [29,31], concentrating them in a minimal volume. The formation of the relevant three-dimensional network can be easily accomplished by employing simple organic chemistry reactions. In particular, it is possible to exploit the nucleophilic reactivity of the CD subunits with double electrophiles such as bis-isocyanates [34], bis-anhydrides [35], or carbonates [36,37]. Alternatively, per-6-halo-6-deoxy-CDs can react with polyamines [38], whereas per-6-azido-6-deoxy-CDs can react with propargyl-calixarenes according to the well-known *CuAAC* (Cu-Catalyzed Azido-Alkyne Coupling) method [33]. Hence, their texture properties [39], the functional mobility of a solvent medium in their pore network [34], and their adsorption abilities can be largely modulated. Moreover, sensitivity to particular conditions such as pH variations can be easily achieved [40], and task-specific systems can even be designed [41,42]. In particular, NSs can also serve as functional supports for nanostructured catalysts [43,44,45,46,47]. In fact, their ability to concentrate an organic substrate near the reactive center may result in a significant activity enhancement. Moreover, the dispersion of the catalyst into the NS matrix can improve its recovery and reuse. We recently succeeded in accomplishing the synthesis of two composite photocatalyst materials where a photoactive species, constituted by silver nanoparticles, dispersed onto reduced graphene oxide (RGOAg), was dispersed (10% *w*/*w*) in two different NS matrixes, made up by either beta-cyclodextrin (βCD) reticulated with hexamethylene-diisocyanate (H) or heptakis-6-iodo-6-deoxy (ICD) reticulated with the *N*-methyl-bis-(3aminopropyl)-amine (A). These materials showed an interesting activity enhancement compared to the plain RGOAg in the photodegradation of some model dyes [28].

As a development of the study mentioned above, here we performed a more systematic investigation on the enhancement effect provided by the NS on the activity of a silver/reduced graphene oxide photoactive species. In particular, we exploited these systems for the degradation of emerging pollutants, paying particular attention to the role of the physicochemical characteristics of the NS support, as they are modulated by the chain extender. 

**Figure 1:**
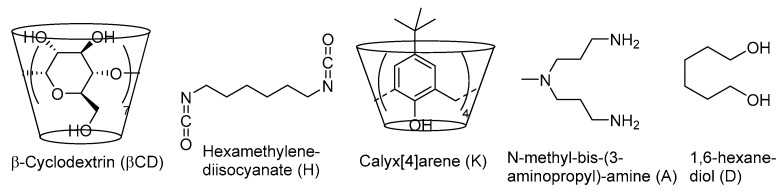
Structures of the NS synthons.

In detail, we considered the reticulation of native βCD with the diisocyanate H in the presence of three suitable chain extenders, namely 1,6-hexanediol (D), *N*-methyl-bis-(3aminopropyl)-amine (A), and *p*-^t-^Bu-calix[4]arene (K, Figure 1). In this way, we focused on the possible outcome of a significant modification in the microscopic properties of the NS matrix (hydrophobic character, presence of pH-sensitive amine groups, etc.). The functional sorbents were loaded with the photoactive species according to two different strategies, i.e., either dispersing a pre-formed RGOAg material in the NS during its formation reaction (*ex-ante* procedure) or dispersing plain graphene oxide (GO) into the matrix, then adsorbing a water-soluble silver salt (AgNO_3_) and eventually reducing with excess sodium borohydride (*ex-post* procedure). In this way, six different composite photocatalysts were obtained (indicated as Cat1–Cat6), the performances of which were compared to those of plain RGOAg. 

**Figure 2:**
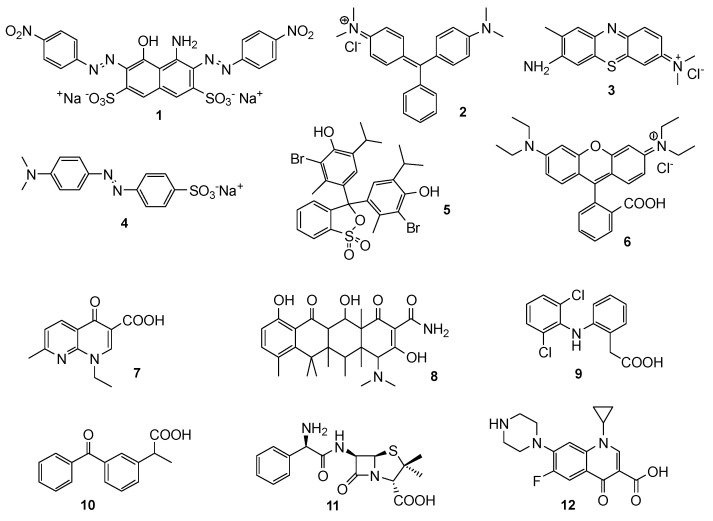
Structures of the model pollutants.

The efficacy of the obtained photocatalysts was tested towards a panel of 12 different diversely structured model organic pollutants (Figure 2), namely 6 dyes (Naphthol blue-black 1, Malachite green 2, Toluidine blue 3, Methyl orange 4, Bromochresol blue 5, Rhodamine B 6) and 6 drugs (Nalidixic acid 7, Tetracycline 8, Diclofenac 9, Ketoprofen 10, Ampicillin 11 and Ciprofloxacin 12).

## 2. Materials and Methods

### 2.1. Materials

All the reagents needed (Cyclolab (Budapest, Hungary), Aldrich (St. Louis, MO, USA), Merck (Rahway, NJ, USA)) were used as purchased, with no further purification. Anhydrous βCD was obtained by drying the commercial hydrate product in vacuo over P_2_O_5_ at 90 °C overnight. The *p*-^t-^Bu-calix[4]arene (K) was prepared according to the procedures reported in the literature. The anhydrous DMF was prepared by equilibrating the commercial product for at least two days with an amount equal to 20% by weight of molecular sieves such as A4, activated in turn by keeping them overnight in vacuo over P_2_O_5_ at 160 °C.

### 2.2. Instrumentation

UV–Vis spectra were recorded on a Beckmann Coulter DU 800 spectrophotometer (Beckman Coulter Inc., Brea, CA, USA) equipped with a Peltier thermostatic apparatus. FTIR spectra were acquired on an Agilent Tech. CARY630FT-IR instrument (Agilent Technologies Inc., Santa Clara, CA, USA). ^13^C(^1^H) CP-MAS solid-state NMR spectra were recorded on a Bruker Advance II 400 MHz spectrometer (Burker Corp., Billerica, MA, USA). Raman spectra were acquired on a Horiba LabRAM HR Evolution apparatus (Horiba Ltd., Kyoto, Japan) using a 532 nm laser source. ICP-OES analyses were performed on a Perkin Elmer Model Optima 2100 apparatus (PerkinElmer Inc., Waltham, MA, USA) equipped with an AS-90 model autosampler. For the quantitative determination of Ampicillin, it was necessary to use an Agilent 7890B HPLC-MS apparatus equipped with a Q-TOF hybrid mass analyzer.

### 2.3. Preparation of GO and Its Derivatives

#### 2.3.1. Pristine Graphene Oxide (pGO)

The pristine pGO product has been prepared according to a modified Hummers procedure. In detail, graphene (1.04 g), NaNO_3_ (0.52 g, 6.1 mmol) and conc. H_2_SO_4_ (23 mL, 429 mmol) are mixed at 0 °C under vigorous stirring, then solid KMnO_4_ (3.02 g, 19.1 mmol) is cautiously added in small portions. The mixture is kept under stirring for 2 h at 0 °C and subsequently warmed at 35 °C for 30 min. Then, H_2_O (46 mL) is added and the system is stirred at 90 °C for 50 min. After a short cooling, further water (140 mL) and 30% H_2_O_2_ (3 mL) are added, and the mixture is allowed to settle at r.t. for 3 days. The precipitated solid is decanted and treated with conc. HCl (20 mL) at r.t. overnight. The solid is separated and repeatedly washed with warm water (70 °C); then, it is suspended again in cold water (110 mL) and cautiously treated with NaHCO_3_ up to neutrality. The final product is centrifuged, washed with methanol (twice) and diethyl ether (twice), dried overnight in vacuo over P_2_O_5_ at 60 °C, and crunched in a mortar. Yield: 1.20 g.

#### 2.3.2. Reduced Graphene Oxide (RGO)

pGO (400 mg) is suspended in water (30 mL) and treated with an excess NaBH_4_ (400 mg, 10.6 mmol). The mixture is allowed to stir at r.t. for 30 min; then, HCl 1 M is added dropwise up to neutrality. The solid is centrifuged, washed with methanol (twice) and diethyl ether (twice), dried overnight in vacuo over P_2_O_5_ at 60 °C, and finally crunched in a mortar. Yield: 395 mg.

#### 2.3.3. Reduced Graphene Oxide/Silver Nanoparticles Composite (RGOAg)

pGO (214 mg) is dispersed in water (15 mL) by brief sonication, and solid AgNO_3_ (339 mg, 2 mmol) is dissolved into the suspension. Then, NaBH_4_ (75 mg, 2 mmol) is added, and the mixture is kept under vigorous stirring at r.t. for 30 min. The system is allowed to settle overnight, then the supernatant liquor is decanted off. The solid is washed with methanol (twice) and diethyl ether (twice) and filtered off. Yield: 401 mg.

### 2.4. Synthesis of Nanosponge Composite Materials and Photocatalysts

#### 2.4.1. NSGO1

The diisocyanate H (320 μL, 336 mg, 2 mmol) and triethylamine (TEA, 140 μL) are dissolved in anhydrous DMF (4.0 mL). Then, pGO (100 mg) is added, the mixture is sonicated for one minute, and heated at 60 °C under magnetic stirring (360 rpm). Anhydrous βCD (567 mg, 0.5 mmol) is subsequently added, and the system is kept at 60 °C under magnetic stirring for 3 h, during which a strong increase in viscosity is observed until a rubbery mass is formed. At this point, stirring is stopped and the system is kept at 60 °C overnight. The crude solid obtained is grossly crunched, transferred into a centrifuge tube with methanol (10 mL), and centrifuged for 5 min at 3800 rpm. The product is again washed with methanol (twice, 20 mL each) and then with ethyl acetate (three times, 20 mL each). The solid residue is left to dry in an oven at 60 °C overnight. Finally, the solid is dried for 24 h in vacuo over P_2_O_5_ at 60 °C, crunched in a mortar, and passed through a 150 μ sieve. Yield: 980 mg.

#### 2.4.2. NSGO2

The diisocyanate H (240 μL, 252 mg, 1.5 mmol), TEA (70 μL), and 1,6-hexanediol (D, 59.0 mg, 0.5 mmol) are dissolved in anhydrous DMF (2.0 mL). Then, pGO (55 mg) is added and the mixture is sonicated for approximately 1 min. The mixture is heated at 60 °C under magnetic stirring; then, βCD (284.0 mg, 0.25 mmol) is added. The reaction system is left at 60 °C for three hours, after which the obtained material is isolated according to the work-up procedure described in the previous case. Yield: 609 mg.

#### 2.4.3. NSGO3

The diisocyanate H (240 μL, 252 mg, 1.5 mmol), TEA (70 μL), and the diol D (59.0 mg, 0.5 mmol) are dissolved in anhydrous DMF (2.0 mL). The solution obtained is placed under magnetic stirring at 60 °C. Then, *p*-^t-^Bu-calix[4]arene (K, 162 mg, 0.25 mmol) is added in small portions over 2 min. The solution is kept under magnetic stirring at 60 °C for 15 min, after which pGO (87 mg) is added, sonicating the mixture for one minute. Subsequently, the system is stirred again at 60 °C, and after three minutes, βCD (284.0 mg, 0.25 mmol) is added. The mixture is left at 60 °C under magnetic stirring and the expected increase in viscosity described previously occurred within 15 min. Stirring is then stopped, and the reaction is allowed to complete overnight still at 60 °C in an oven. The raw material is isolated and purified as described hereinabove. Yield: 760 mg.

#### 2.4.4. NSGO4

The diisocyanate H (320 μL, 336 mg, 2 mmol) is dissolved in anhydrous DMF (4.0 mL), and the triamine A (160 μL, 144 mg, 1 mmol) is slowly added under magnetic stirring (360 rpm). The mixture obtained is heated at 60 °C for 1 h, then pGO (116 mg) is added and the mixture is sonicated for 1 min. Subsequently, the system is heated again at 60 °C, and after 3 min, βCD (567 mg, 0.5 mmol) is added. The mixture is left at 60 °C under magnetic stirring until the formation of a rubbery mass occurs, and the reaction is allowed to complete overnight still at 60 °C. The raw material is isolated and purified as described hereinabove. Yield: 987 mg.

#### 2.4.5. Cat1

The synthetic procedure is identical to that described for the NSGO2 material, but for the fact that RGOAg (102 mg) is added instead of pGO. Yield: 747 mg.

#### 2.4.6. Cat2

AgNO_3_ (63 mg, 0.4 mmol) is dissolved in methanol (2 mL), and the NSGO2 material (400 mg) is added to the solution. The suspension is allowed to stir for 1 h in an orbital stirrer and then dried in vacuo. The solid obtained is resuspended in 2 mL of distilled water, and excess NaBH_4_ (76 mg, 2 mmol) is added to the suspension under magnetic stirring for 30 min. The solid product is centrifuged, washed as described for the previous materials, and finally dried. Yield: 360 mg.

#### 2.4.7. Cat3

The synthetic procedure is identical to that described for the NSGO3 material, but for the fact that RGOAg (194 mg) is added instead of pGO. Yield: 946 mg.

#### 2.4.8. Cat4

AgNO_3_ (120.5 mg, 0.71 mmol) is dissolved in methanol (4 mL), and the NSGO3 material (725 mg) is added to the solution. The suspension is allowed to stir for 1 h in an orbital stirrer and then dried in vacuo. The solid obtained is re-suspended in 2 mL of distilled water, and excess NaBH_4_ (152 mg, 4 mmol) is added to the suspension under magnetic stirring for 30 min. The solid product is centrifuged, washed as described for the previous materials, and finally dried. Yield: 725 mg.

#### 2.4.9. Cat5

The procedure is identical to that described for the NSGO4 material, but for the fact that RGOAg (187 mg) is added instead of pGO. Yield: 645 mg.

#### 2.4.10. Cat6

AgNO_3_ (95 mg, 0.60 mmol) is dissolved in methanol (3 mL), and the NSGO3 material (753 mg) is added to the solution. The suspension is allowed to stir for 1 h in an orbital stirrer and then dried in vacuo. The solid obtained is re-suspended in 2 mL of distilled water, and excess NaBH_4_ (119 mg, 3 mmol) is added to the suspension under magnetic stirring for 30 min. The solid product is centrifuged, washed as described for the previous materials, and finally dried. Yield: 753 mg.

### 2.5. ICP Analyses

A carefully weighed amount (ca. 10.00 mg) of each silver-containing material was placed in a small ceramic crucible and mineralized with conc. HNO_3_ at 90 °C up to complete decomposition. The residue was dissolved in freshly double-distilled water and diluted up to 100 mL. The obtained solution was filtered through a 0.45 μm Millipore membrane before analysis.

### 2.6. Preliminary Adsorption Tests

Aqueous stock buffer solutions were prepared as follows: pH 4.4: 200 mg of CH_3_COONa were dissolved in 250 mL of freshly double-distilled water, then the proper amount of HCl 1 M was added to reach the desired pH value; pH 6.7: 232 mg of NaH_2_PO_4_ and 113 mg of Na_2_HPO_4_ were dissolved in 250 mL of freshly double-distilled of water. The actual pH of all the buffer stock solutions was checked by a CRISOM MicropH 2001 instrument equipped with a commercial glass electrode. Buffers, in turn, were used to prepare stock solutions of the model pollutants, at the following concentrations: 40 μM for 1, 4, 5, 7, 8, 9, and 12; 25 μM for 10; 20 μM for 2, 3 and 11; 10 μM for 6.

Samples for the preliminary adsorption tests were prepared by mixing a fixed volume of guest solution (2.0 mL) with a carefully weighted amount (4.00 ± 0.05 mg) of each NSGO1–NSGO4 material. The samples were shaken at r. t. for 90 min, and then, centrifuged for 15 min at 4000 rpm. The supernatant liquor was carefully pipetted after centrifugation; then, the percent amount of guest left in solution at equilibrium was simply estimated using UV–Vis spectrophotometry, comparing the absorbances of the starting and final solutions.

### 2.7. Photodegradation Experiments

The proper amount of photocatalyst (namely 2.00 ± 0.05 mg in the case of RGOAg, 14.20 ± 0.05 mg for Cat1, 10.00 ± 0.05 mg for Cat2–Cat6) is mixed in a vial with a solution (10 mL) of the organic substrate at the proper concentration, namely 40 μM for substrates 1, 4, 5, 7, 8, 9 and 12; 25 μM for 10; 20 μM for 2, 3 and 11; 10 μM for 6. The sample is allowed to equilibrate in the dark for 30 min; then, it is irradiated in a homemade apparatus made by a box, having the internal walls covered in tin foil, and a 50 W halogen lamp placed in such a way to provide an irradiation power as large as 275 W/m^2^. Irradiation is maintained for 2 h while stirring is achieved by mounting the described apparatus on an orbital shaker plate. Then, the sample is centrifuged (6 min, 3000 rpm) to separate the photocatalyst, and finally, the residual amount of substrate is determined by UV–Vis spectrophotometry. In order to gain the correct estimation of the catalytic activity, the amount of unreacted adsorbed dye was evaluated. The catalyst recovered from the photodegradation experiment is repeatedly washed with small portions (1 mL each) of methanol till washings appear colorless (three or four passages are usually sufficient; at each passage, the photocatalyst is recovered by centrifugation at 3000 rpm for 6 min). The collected liquors are diluted up to 10 mL, and the obtained solution is analyzed by UV–Vis spectrophotometry to determine the residual dye concentration. Finally, the overall amount of unreacted dye is obtained by trivial stoichiometric calculations. 

For the kinetic tests, eight equal samples are prepared by placing in a vial a given amount of photocatalyst (2.00 ± 0.02 mg) and substrate solution (5 mL in the case of RGOAg, 2 mL in the case of Cat5 and Cat6). The vials are allowed to equilibrate as described above and then placed in the photoreactor. Once irradiation has started, the various samples are taken at fixed times (15, 30, 45, 60, 90, 120, 180, and 240 min), to be analyzed as described above. From the percentage data of the degraded substrate vs. time, the kinetic curve is then constructed, which is finally subjected to regression analysis. 

For the reuse tests, the photocatalyst recovered from the previous experiments was dried in vacuo at 60 °C overnight, weighed, and re-suspended in a suitable amount of fresh substrate stock solution, in such a way as to keep constant the catalyst-to-solution (*w*/*v*) ratio.

## 3. Results and Discussion

### 3.1. Synthesis

The synthesis of the NS materials (described in detail in the previous section) relies on well-assessed procedures [29,31]; therefore, only a few comments are needed here. In brief, the electrophilic isocyanate groups of the linker H easily react with the nucleophilic -OH groups of the βCD or the K synthons (affording aliphatic or aromatic urethane groups, respectively) as well as with the -OH groups of the diol D chain extender (again, affording aliphatic urethane groups) or with the -NH_2_ groups of the triamine A chain extender (this time affording urea groups). Notably, in the synthesis of materials NSGO3 and Cat3, the order by which the reactants were mixed was chosen in such a way as to ensure that the K and H components had effectively interacted before adding the βCD component. It is also worth noting that possible -OH groups present in either pGO or RGOAg might in principle react and participate in the network formation process. In this way, the carbonaceous material would not be simply embedded, but rather chemically bound to the reticulated network structure. Unfortunately, it is hardly possible to obtain any positive evidence about the occurrence of this process. This, however, would indeed have scarce importance in affecting the possible photocatalytic performances. Rather, the absence of any trace of the carbonaceous component in the washing liquors accounts for its complete incorporation into the NS network. We considered it particularly interesting to verify the outcome of the different procedures for silver loading, in particular for polyamine-decorated materials Cat5 and Cat6. In fact, it had been previously shown that polyamine-based NSs are able to adsorb significant amounts of Ag^+^ ions (up to 20% *w*/*w*, and even more), and effectively stabilize silver nanoparticles [38]. Therefore, the *ex-post* procedure had already been proven successful for silver loading. However, it cannot be taken for granted that it leads to materials in which Ag nanoparticles are necessarily bound onto the graphene constituent. In the present case, we planned our syntheses in such a way as to gain a theoretical 10% silver loading in the NS-based photocatalysts. 

### 3.2. Characterization

Spectroscopic characterization of the materials was essentially carried out by ATR-FTIR spectroscopy and the ^13^C(^1^H) CP-MAS Solid State NMR technique. These techniques allowed us to unambiguously identify the signals expected for the various functional groups formed in the reticulation reactions, and the typical fingerprints of the moieties from the constituting synthons (cyclodextrins, calixarenes, linkers). Then, the amount of silver present was determined by ICP techniques.

**Figure 3:**
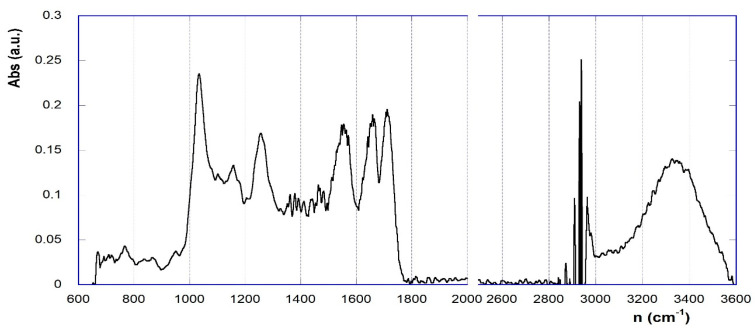
FT–IR spectrum of Cat3 (after baseline correction).

In general, the FT-IR spectra (as an exemplificative example, the spectrum of Cat3 is depicted in Figure 3) show the -OH stretching band centered at ca. 3370 cm^−1^, due to the presence of the βCD synthon, whose typical fingerprint band system can also be easily spotted out in the 1300–1000 cm^−1^ region. The fingerprint area of the spectrum does not allow the unambiguous identification of the signals relating to the calixarene constituent, as they are superimposed on other signals. Similarly, possible signals from the graphene component can hardly be detected. Conversely, the most interesting section of the spectrum is the region 1750–1500 cm^−1^, where the Amide-I-like and Amide-II-like signals of the urethane and urea groups can be found. The presence of at least two signals in this region undoubtedly accounts for the actual occurrence of the cross-linked nanosponge network. These signals can possibly be subjected to deconvolution analysis in order to detect the contributions of the different groups possibly present. The relevant attributions are summarized in Table 1. Indeed, the recognition of the three different groups provides positive evidence of the incorporation of the A or K chain extender in the relevant materials. 

**Figure 4:**
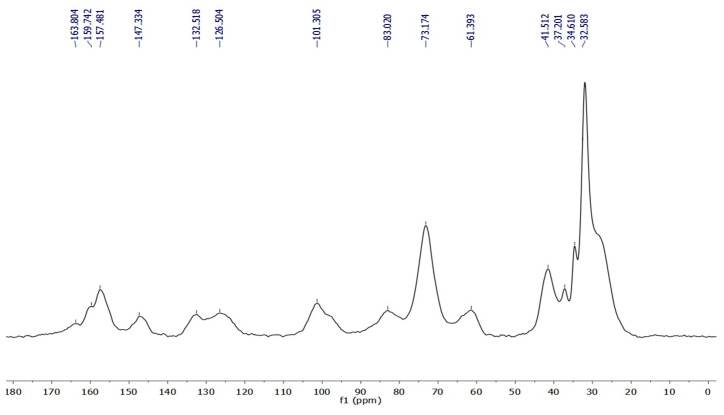
^13^C[^1^H] CP-MAS NMR spectrum of Cat3 (after baseline correction).

The ^13^C(^1^H) CP-MAS solid-state NMR spectra enable unambiguously identifying the actual presence of the possible calixarene component in materials NSGO3, Cat3, and Cat4, thanks to the presence of the relevant signals in the aliphatic and aromatic carbon regions. Complete signal attributions are summarized in Table 2 (the spectrum of Cat3 is depicted in Figure 4 for exemplificative purposes). In detail, along with the expected signals for the βCD subunits, spectra of the latter materials also clearly feature the typical intense band in the aliphatic C region (*^t-^*butyl and methylene C atoms) and a system of four signals in the aromatic C region. In addition, the spectra of all materials feature the signals relevant to the linker and chain extender units in the aliphatic C spectral region, together with a signal cluster centered around 160 ppm in the urethane/urea carbonyl region (which, again, accounts for the actual formation of the polymer network). Notably, the graphene carbonaceous component does not provide signals of detectable intensity. This finding can be partly explained by the fact that it is indeed only a minor component of the composite. Moreover, it must also be taken into account that the CP-MAS technique relies on the enhancement of the C signals due to the cross-polarization with the ^1^H nuclei present in the sample. Thus, the ^13^C nuclei of the graphene component, which are not bound to any H atom, can hardly benefit from this effect.

**Figure 5:**
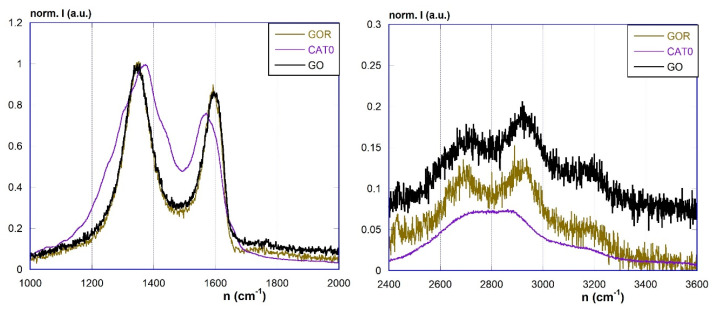
Raman spectra of pGO, RGO, and RGOAg in the 1000–2000 cm^−1^ (**left**) and 2400–3600 cm^−1^ (**right**) regions.

**Figure 6:**
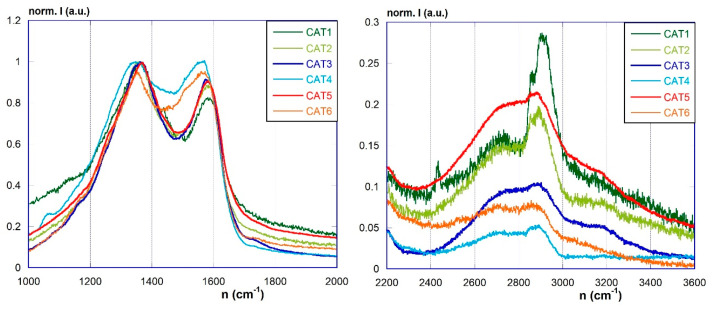
Raman spectra of Cat1–Cat6 in the 1000–2000 cm^−1^ (**left**) and 2400–3600 cm^−1^ (**right**) regions.

More convincing evidence of the actual presence of the graphene component in the materials can be obtained by Raman spectroscopy. Raman spectra of the materials pGO, RGO, and RGOAg are depicted in Figure 5, whereas those of the photocatalyst materials Cat1–Cat6 are in Figure 6. In general, the spectra feature the well-known systems of bands in the ranges 1000–1800 cm^−1^ (given by the superimposition of the D, D″, D*, D′, and G bands) and 2400–3400 cm^−1^ region (with the 2D and S3 bands). It is well known that the amount of structural defects and the extent of the graphene-like domains, as well as the number of graphene layers, can be related by the intensity ratios between the D and G bands (*I*_D_/*I*_G_), the 2D and G bands (*I*_2D_/*I*_G_), and the 2D and S3 bands (*I*_2D_/*I*_S3_) [48,49,50,51,52,53]. These ratios can be obtained after the proper deconvolution analyses. The reduction of pGO to RGO causes only small variations in the shape of the spectrum, and the deconvolution analysis of the signals shows a slight variation of the above ratios. Considering RGOAg material, it is possible to note that the introduction of silver nanoparticles causes modifications in the spectrum. The band system in the 1000–1800 cm^−1^ region shows a shift in the position of the maximum and the appearance of several shoulders. The deconvolution analysis indicates in particular a shift to higher wavelength numbers of the D band (at 1371 cm^−1^ approx.) and the occurrence of three new bands (1256, 1361, 1437 cm^−1^). At the same time, the 2400–3400 cm^−1^ region also shows a shift of the 2D and S3 bands and a substantial loss of resolution. These observations indicate the existence of strong interactions in the composite material between the metallic and carbon components, which undergo significant structural modifications. 

On passing to the spectra of Cat1–Cat6 materials, the concomitant presence of the overwhelming NS component makes the analysis much more complicated, because of the superimposition of the signals relevant to the organic matrix to those of the carbonaceous component. The spectra appear poorly resolved even in the 1000–1800 cm^−1^ region and the signals are in any case displaced compared to the characteristic position of the pristine pGO. There is also the complete loss of resolution of the 2400–3400 cm^−1^ area, where the graphene band system is no longer clearly distinguishable because intense signals are even visible around 2900 cm^−1^ attributable to the aliphatic C-H stretching. Therefore, deconvolution analysis of the spectral signals becomes pointless. Nevertheless, spectra undoubtedly provide convincing evidence of the actual presence of the graphene component embedded within the NS network, and of their interaction as well.

The photocatalysts were characterized by ICP analysis, in order to determine their actual Ag loadings. Results are summarized in Table 3. Notably, RGOAg shows a loading close to the theoretical one of 50%, in agreement with what is reported in the literature. For materials Cat1–Cat6, silver loadings between 5% and 9% were found, which are lower than the theoretical 10% expected. It is noteworthy that the materials that best retain the Ag component are Cat5 and Cat6, i.e., those decorated with the amine chain extender (consistent with the ability of amine ligands to effectively coordinate the Ag^+^ ion). The ex-ante loading procedure leads to significant metal loss, which can be justified by possible metal leaching during the work-up procedures. The ex-post loading procedure appears poorly effective in the case of Cat2, probably due to a lack of strong binding sites. Conversely, the ex-post loading of Cat4 works much better, probably because of the favorable interaction between the soft Lewis acid Ag^+^ cation with the calixarene component cavities. 

**Figure 7:**
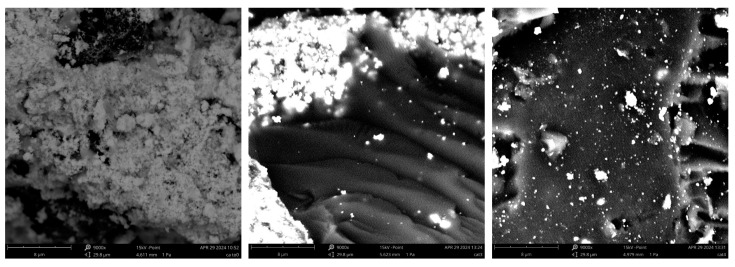
Representative SEM micrographs of RGOAg (**left**), Cat3 (**center**), and Cat4 (**right**).

Finally, morphological characterization was performed using SEM techniques. Some representative micrographs (namely for RGOAg, Cat3, and Cat4) are shown in Figure 7 for exemplificative scope (the complete micrographs are collected in Supplementary Materials, Figure S1). 

Pristine RGOAg shows a dense covering of tiny silver particles. The materials obtained with the *ex-ante* silver loading strategy (after having been crushed in a mortar and sieved at 150 μ) appear as compact masses with a quite clean surface, over which few irregular Ag particles are present (roughly several tenths or even few μ in size, as it can be spotted out by inspection of SEM images), but for those rare regions where the enclosed RGOAg component is occasionally exposed. This finding suggests that a small part of the silver particles formerly present in the RGOAg component could have been mechanically detached from the carbonaceous support during the polymer network synthesis. Conversely, the presence of tiny surface silver particles (most of which appear smaller than 0.3 μ, from inspection of SEM images) is much more apparent for the materials prepared with the *ex-post* loading strategy, supporting the hypothesis that, in the latter cases, Ag particles are not forced to be bound to the carbonaceous support embedded into the NS network. As a final remark, it is interesting to notice the materials containing the calixarene chain extender (Cat3 and Cat4) appear as relatively smooth masses, whereas the other NSs appear to have a much more wrinkled surface. This can be tentatively explained considering that the use of the calixarene moiety, bearing four nucleophilic –OH groups, enables obtaining a more reticulated network.

### 3.3. Preliminary Adsorption Tests

Before studying the photocatalytic properties of Cat1–Cat6 materials, a preliminary assessment of the intrinsic adsorption abilities of these NS–graphene composite matrices was needed, for understanding the effects from the presence of the chain extender component. For this purpose, four model composite materials, NSGO1–NSGO4, each loaded with 10% pGO (*w*/*w*), were prepared. In detail, NSGO1 features the diisocyanate H crosslinker only; in NSGO2, diol D as chain extender is present (as in Cat1 and Cat2); in NSGO3, the calixarene K is present (as in Cat3 and Cat4); finally, NSGO4 contains the triamine A (as in Cat5 and Cat6). The adsorption tests were carried out at pH 4.4 and 6.7 (in analogy with some previous works) in order to verify the possible effect of the pH medium, which could be particularly interesting in the case of the potentially pH-sensitive material NSGO4. The dyes 1–6 only were considered, because they are far more viable to quantify by UV–Vis spectrophotometry. The obtained results are summarized in Table 4. 

Collected data generally indicate a good affinity of the various materials towards the substrates, with few exceptions. The material that presented the best average adsorption ability was the non-functionalized NSGO1 (69%), followed by NSGO2 (65%) containing the aliphatic D chain extender. The presence of the calixarene component in NSGO3 has a rather negative effect, as the average percentage drops down to 41%. The amino chain extender of NSGO4 also has a negative effect, but in this case, it is important to take into account the pH value too. In fact, due to the presence of the tertiary amine groups, which are partly protonated even under neutral pH conditions, the nanosponge backbone is positively charged. This enhances the electrostatic effects affecting the adsorption properties of the material. Consistently, it is observed that the average adsorptions rise from 36% at pH 4.4 to 56% at pH 6.7. Furthermore, the significant decrease in affinity for NSGO3, particularly in the case of the dyes 1, 4, 5 and 6 (namely, Naphthol blue-black, Methyl orange, Bromochresol green and Rhodamine B), is not trivial, because the presence of K, by increasing the hydrophobic character of the material, should favor effective van der Waals interactions with the large conjugated structure of the organic guests. However, it must be taken into account that these dyes have anionic sulfonate or carboxylate groups, involving the presence of a localized charge. The charged groups interact effectively with the aqueous solvent, overall causing a decrease in affinity for the highly hydrophobic NS backbone. Indeed, it should also be kept in mind that an increase in the hydrophobic character makes the material less permeable to the aqueous solvent medium. Conversely, for those dyes such as 2 and 3 (Malachite green and Toluidine blue), carrying a positive charge largely delocalized over their conjugate structure, the increase in the hydrophobic character of the material has a limited outcome on their possible adsorption. 

### 3.4. Photocatalytic Activity

The experimental procedures for performing the photodegradation tests are reported in the Materials and Methods section. A few comments should be reported herein. The tests rely on the simple idea of comparing the UV–Vis absorption of the pollutant solution before and after the irradiation in the presence of the photoactive material. The experimental conditions (irradiation power and time) were chosen in analogy with our previous work [28]. The initial amounts of the different substrates were not the same (see Section 2.7), but were suitably chosen in such a way to optimize their UV–Vis detection. It is important to stress that, in order to accomplish a correct evaluation of the catalytic activity, it is mandatory to rule out any contribution to the decrease in the solution absorbance deriving from the mere adsorption of the substrate onto the material. Hence, elution of the recovered photocatalyst with a suitable solvent (and evaluation of the substrate concentration in the eluate) is needed. Moreover, the amounts of photocatalysts in the various tests were chosen in such a way as to have roughly the same amount of silver in each test sample. In fact, all the results obtained, in terms of the mole amount of degraded organic substrate, must be normalized for the silver content in order to obtain a correct evaluation of the catalytic efficiency. Finally, the normalized results for the composite catalysts Cat1–Cat6 were compared with those for the plain photoactive species RGOAg, in order to ascertain the effect of the NS matrix on the catalytic efficiency. This effect is suitably quantified as the ratio between the normalized degradation yields for each composite catalyst and the corresponding datum for RGOAg. The obtained results are collected in Table 5, Table 6 and Table 7 (data are also illustrated in in Supplementary Materials, Figure S2).

All the photocatalysts generally show good activities, although strong differences were observed between different substrates. Considering the normalized degradation yields, dyes were degraded more efficiently than drugs, on average. In particular, dyes Naphthol blue black 1, Malachite green 2, and Toluidine blue 3 show the best degradation yields. By contrast, Rhodamine B 6 appears more resistant. On passing to drugs, fair yields can be found for Nalidixic acid 7, Tetracyclin 8, and Ciprofloxacin 12. Conversely, Diclofenac 9 and Ketoprophene 10 appear particularly resistant to photodegradation. As far as the latter two drugs are concerned, it is noteworthy that polyamine-decorated materials Cat5 and Cat6 appear to be the only photocatalysts able to promote degradation up to a fair extent, whereas even plain RGOAg has no significant effect. Because of this, it was not possible to quantitatively assess any activity enhancement due to the NS matrix for these two substrates.

Considering the effect of the NS matrix, a close inspection of data in Table 7 reveals that, significant activity enhancement (>1.2) can be found in 33 cases out of 59 (24 out of 36 if we consider dyes only; up to 9 times, in the case of 5 with Cat1), whereas significant activity decrease (<0.8) occurs in 11 cases out of 60 (8 out of 23, on considering drugs only). As far as the average behavior of the different materials is concerned, the best performances were shown by amine-decorated materials Cat5 and Cat6 (2.3 both). Satisfactory performances were shown also by Cat1 (2.2) and Cat2 (2.1), bearing the aliphatic chain extender D. Conversely, only a fair average enhancement (1.5) was found for the calixarene-decorated material Cat3, whereas no significant average matrix effect occurred for Cat4. In perfect analogy with the preliminary adsorption tests, these data indicate that the large increase in the hydrophobic character caused by the presence of the calixarene component has a negative outcome on the overall properties of the composite material. Notably, only in this case, the different protocol used for silver loading has a significant impact on the photocatalytic performances, in agreement with the well-known reactivity–selectivity principle. Otherwise, the use of either the ex-ante or the ex-post silver loading method provides no particular advantage. 

On passing to analyze the collected data as a function of the organic substrate, the largest average enhancement can be found for dyes 5 (5.6), 1 (3.1), and up to a lesser extent, 4 (2.4). Conversely, among drugs, significant activity enhancement can be found only for 11 (2.0; a modest average enhancement of 1.3 can be found also for 12). By contrast, no average enhancement can be found for 6, 7, and 8. These results appear quite tricky to rationalize because no correlation is apparent with the structure of the substrates, nor with their average affinity for the NS matrix as obtained from the preliminary adsorption tests. Indeed, it should be kept in mind that guest affinity for nanosponges is the outcome of a fine balance between different and mutually contrasting effects (specific and unspecific host–guest interactions, solvation effects, etc.). In the present case, the situation is complicated by the possible concomitant occurrence of further specific interaction with the graphene and the silver nanoparticle components. Therefore, any attempt to actually predict the overall outcome of all these factors would be a desperate task.

Data relevant to degradation percentages should be compared with those reported in the literature. A massive amount of experimental work has been carried out on the photodegradation of both dyes and drugs. Recent works on the subject are virtually countless, indeed, and are periodically reviewed [11,12,13,14,15,16,17,18,19,20,21,22,23,24,25,26,54,55,56]. Plenty of papers make a claim for very satisfactory (>90%) [10,57,58,59] or even almost complete (>97%) [60,61,62,63,64,65] apparent removal of all the substrates considered herein. Again, it is important to stress that, unlike most reports, our data take into account the correction for the amount of undegraded substrate possibly adsorbed onto the photoactive material. We always observed that the catalyst recovered after each photodegradation test, releasing significant amounts of unreacted substrate (particularly in the case of dyes) after washing with methanol. Just to cite a typical case, dye 6 shows a very satisfactory 90% apparent removal with title RGOAg, as it can be simply evaluated from the absorbance of the aqueous solution after the irradiation experiment. However, this result reduces to a modest 18% once the unreacted dye has been washed away from the catalyst. Therefore, our results are hardly comparable with those present in previous works.

The possible recyclability of the materials was verified. In particular, their possible reuse in the degradation of dye 3, as a model substrate, was tested for five catalytic cycles. Unfortunately, the obtained results (collected in Table 8) were not satisfactory. In fact, a significant drop in activity could be observed after the first cycle. Then, activity was roughly constant after the third cycle for Cat1–Cat3 and Cat5, whereas it decreased almost to zero for Cat2 and Cat4. These findings can be attributable to the loss of metal loading during the process. Once again, it was possible to note that the *ex-post* procedure appears disadvantageous. 

Finally, the photodegradation kinetics were studied in order to obtain further insights into the kinetic and mechanistic course of the process. For this purpose, RGOAg, Cat5, and Cat6 were selected (the first as the reference, the other two as the best-performing systems on average). As model substrates, dyes 3 and 4 and drug 8 were considered. The data collected, summarized in Table 9, show some unexpected features. In fact, in some cases, the degradation followed a first-order kinetic, whereas in other cases, the data trend can be mathematically modeled using a “stretched exponential” function of the following type:

P(t) = P_∞_·(1 − exp [−(*k*t)*^n^*])a = 1, (1)

where P represents the percent of the degraded substrate, *k* is the apparent kinetic constant, and the exponential “stretching” parameter *n* accounts for the deviation of the kinetic course from the standard first-order trend (*n* values range between 0 and 1; for *n* = 1 an ordinary first-order kinetic occurs). Kinetic trends that can be described using this type of mathematical law (reported also as the Kohlrausch equation) have been occasionally reported in the literature [66,67,68]. The physicochemical interpretation of this type of dependence is still currently under debate. However, the observation of this trend has been generally justified claiming the occurrence of some “inhomogeneity” in the behavior of the catalytic system. In other words, the observed behavior cannot be assimilated to that of a “simple” system in solution, but it should rather be seen as deriving from the superimposition of a “continuum” of microsystems, described by a suitable distribution function. This hypothesis rules out the idea that the photodegradation process simply occurs through the interaction between the substrate and free ROS species in solution. Rather, it suggests that a direct interaction between the substrate and the surface of the photocatalyst can play an important role. It is important to notice that this behavior was observed, in particular, with dyes 3 and 4, having a highly conjugated molecule able to interact with the graphene material, but not with the more hydrophilic tetracycline 8. This behavior, combined with the fact that dyes absorb visible radiation (produced by the light source) more effectively than drugs, could even suggest that the degradation process involves the substrate in its electronically excited state, rather than in the fundamental state. Finally, it is worth noting that, in the case of dyes, apparently there was no direct correspondence between the values of the apparent kinetic constants *k* and those of the degradation percentages at 2 h reported previously in Table 5. In the case of 3, this apparent anomaly is fully justified by the different values of the parameter *n*. Moreover, in the case of 4, the reaction seems to proceed to completion only with Cat6, whereas the regression parameters indicate that the degradation seems to stop at 26% with RGOAg and 46% with Cat5. Also, for Tet with RGOAg, the regression indicates a maximum degradation percentage of 52%. The last observations do not appear easy to rationalize, and they will be subjected to detailed future mechanistic studies, which were indeed by far beyond the scope of the present work.

## 4. Conclusions

In summary, the possibility was positively assessed to employ composite photocatalytic materials obtained by associating a silver-loaded reduced graphene oxide photoactive species into a nanosponge matrix. These materials were suitably characterized by the FT-IR, CP-MAS NMR, Raman, and SEM techniques. In particular, the possible outcome of the presence of a suitable chain extender in the design of the hyper-crosslinked organic sorbent support was addressed. In a significant number of cases (in particular, as far as the degradation of dyes is concerned), the presence of the nanosponge matrix can enhance the catalytic performances (up to nine times). Data show that the photocatalyst performances are favorably affected by the presence of a polyamine component, which also provides pH sensitivity to the material. Conversely, association with a calixarene host seems unfavorable, likely due to the large increase in the hydrophobic character of the material. From a methodological standpoint, it is important to stress that the results presented herein have been suitably corrected for the adsorption of the photoactive material, thus providing a reliable evaluation of the true degradation efficacy of the photodegradation system. In perspective, the results presented in this work appear quite promising, and might be helpful in the design of suitable devices for wastewater treatment, specifically devoted to the elimination of emerging organic pollutants. 

## Figures and Tables

**Table 1:** Attribution of the Amide-I- and Amide-II-type signals in NSs.

Group	Amide-I (cm^−1^)	Amide-II (cm^−1^)
Alkyl urethane	1640–1700	1530–1545
Aryl uretane	1710–1730	1520–1535
Dialkyl urea	1620–1630	1575–1585

**Table 2:** Attribution of the Amide-I- and Amide-II-type signals in NSs.

Signal Attribution		ppm
βCD	C(1)	100~105
	C(2,3,5 cumulative)	70~75
	C(4)	81~84
	C(6)	60~65
K	*^t-^*butyl	31~33 (CH_3_-); 34~35 (quaternary)
	-CH_2_-	32~33
	C aromatic	125~128; 131~136; 146~147; 155~157
D, H, A	-CH_2_- (not bound to O, N)	26~31
	-CH_2_- (bound to O, N)	40~60
	urethane, urea	158~164

**Table 3:** Silver loadings ^1^ onto materials RGOAg and Cat1–Cat6.

Material	RGOAg	Cat1	Cat2	Cat3	Cat4	Cat5	Cat6
Ag load % *w*/*w*	47	6.7	5.0	6.2	8.5	9.0	9.0

^1^ All data are given within a ±0.3% error.

**Table 4:** Percent adsorption ^1^ for dyes 1–6 into materials NSGO1–NSGO4.

Material	pH	1	2	3	4	5	6
NSGO1	4.4	67	60	88	87	74	72
NSGO2		69	75	88	87	67	40
NSGO3		18	63	81	50	25	9
NSGO4		67	21	21	71	20	27
NSGO1	6.7	63	88	91	79	61	65
NSGO2		53	84	87	69	73	33
NSGO3		12	81	77	32	15	17
NSGO4		74	73	32	95	51	24

^1^ Averages over 3 determinations; all data are given within a ±3% error.

**Table 5:** Percent of photodegraded substrate ^1^.

Material	1	2	3	4	5	6	7	8	9	10	11	12
RGOAg	21	78	59	10	5	18	18	40	0	0	8.5	30
Cat1	46	81	67	6	32	12	12	25	0	0	12	27
Cat2	39	65	63	8	16	10	32	21	0	0	3.5	28
Cat3	30	75	68	9	26	19	21	15	0	0	4.5	40
Cat4	7	85	62	10	6	13	3	14	0	0	-	32
Cat5	94	88	80	37	28	19	10	28	4	10	31	18
Cat6	94	87	72	57	23	12	2	46	13	6	22	23

^1^ Averages over 3 determinations; all data are given within a ±3% error.

**Table 6:** Normalized photodegradation yields (μmoles of photodegraded substrate per mg of Ag co-catalyst).

Material	1	2	3	4	5	6	7	8	9	10	11	12
RGOAg	0.89	1.66	1.26	0.43	0.21	0.19	0.77	1.70	0.00	0.00	0.18	1.28
Cat1	2.75	2.42	2.00	0.36	1.91	0.18	0.72	1.49	0.00	0.00	0.36	1.61
Cat2	3.12	2.60	2.52	0.64	1.28	0.20	2.56	1.68	0.00	0.00	0.14	2.24
Cat3	1.41	1.76	1.60	0.42	1.22	0.22	0.99	0.71	0.00	0.00	0.11	1.88
Cat4	0.46	2.79	2.03	0.66	0.39	0.21	0.20	0.92	0.00	0.00	-	2.10
Cat5	4.18	1.96	1.78	1.64	1.24	0.21	0.44	1.24	0.18	0.28	0.69	0.80
Cat6	4.18	1.93	1.60	2.53	1.02	0.13	0.09	2.04	0.58	0.17	0.49	1.02

**Table 7:** Enhancement of the photocatalytic activity due to the NS matrix.

Material	1	2	3	4	5	6	7	8	11	12
Cat1	3.1	1.5	1.6	0.8	9.0	0.9	0.9	0.9	2.0	1.3
Cat2	3.5	1.6	2.0	1.5	6.0	1.0	3.3	1.0	0.8	1.8
Cat3	1.6	1.1	1.3	1.0	5.8	1.2	1.3	0.4	0.6	1.5
Cat4	0.5	1.7	1.6	1.5	1.8	1.1	0.3	0.5	-	1.6
Cat5	4.7	1.2	1.4	3.9	5.8	1.1	0.6	0.7	3.8	0.6
Cat6	4.7	1.2	1.3	6.0	4.8	0.7	0.1	1.2	2.7	0.8

**Table 8:** Percent of photodegraded substrate 3 in the recycling tests ^1^.

Material	Cycle 1	Cycle 2	Cycle 3	Cycle 4	Cycle 5
RGOAg	59	47	15	18	17
Cat1	67	51	17	12	26
Cat2	63	34	17	17	15
Cat3	68	27	20	24	20
Cat4	62	44	16	18	4
Cat5	80	54	17	27	14
Cat6	72	13	14	11	0

^1^ Averages over 3 determinations; all data are given within a ±3% error.

**Table 9:** Kinetic results.

Material	Substrate	P_∞_	*k* (10^4^ s^−1^)	*n*	Dec.% after 2 h
RGOAg	3	100	1.30 ± 0.08	0.84 ± 0.06	59
Cat5	3	100	1.93 ± 0.08	1	80
Cat6	3	100	1.30 ± 0.08	0.71 ± 0.09	72
RGOAg	4	26 ± 2	4.4 ± 0.5	1	10
Cat5	4	45 ± 2	4.4 ± 0.4	0.77 ± 0.08	37
Cat6	4	100	0.91 ± 0.13	0.55 ± 0.05	57
RGOAg	8	52 ± 2	2.0 ± 0.2	1	40
Cat5	8	100	1.10 ± 0.05	1	28
Cat6	8	100	2.0 ± 0.1	1	46

## Data Availability

Dataset available upon request from the authors.

## Supplementary Materials

The following supporting information can be downloaded at: https://www.mdpi.com/article/10.3390/ma17102319/s1, Figure S1: SEM Micrographs; Figure S2: Histograms for photodegradation data.
