# Supplementary material for: Composite RGO/Ag/Nanosponge Materials for the Photodegradation of Emerging Pollutants from Wastewaters
Source: Materials (Basel). 2024 May 14;17(10):2319. doi: 10.3390/ma17102319 (PMC11123357; doi:10.3390/ma17102319)

# Composite RGO/Ag/nanosponge materials for the photodegradation of emerging pollutants from wastewaters

**Ettore Madonia [1], Antonella Di Vincenzo [2,*], Alberto Pettignano [3], Roberto Scaffaro [4], Emmanuel Fortunato Gulino [4], Pellegrino Conte [1] and Paolo Lo Meo [2,*]**

Supporting Information

Figure S1. SEM Micrographs.

Figure S2. Histograms for photodegradation data.

**Figure S1. SEM Micrographs.**

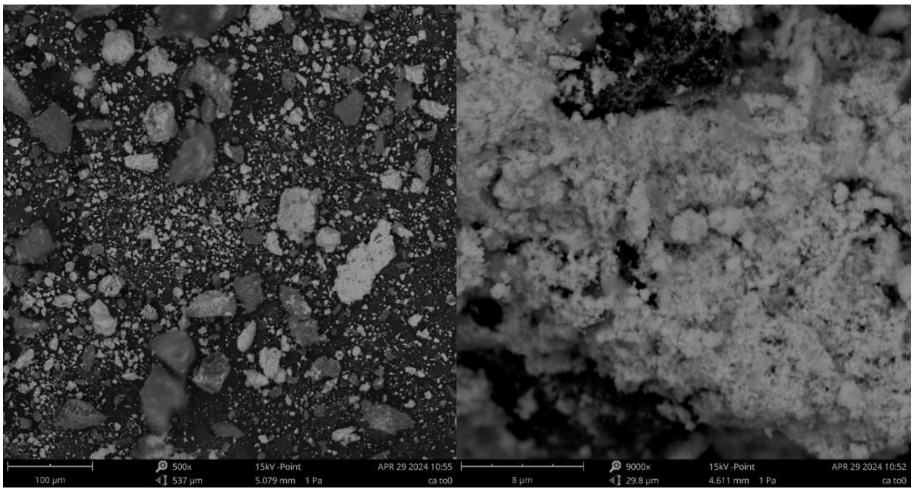


**RGOAg**

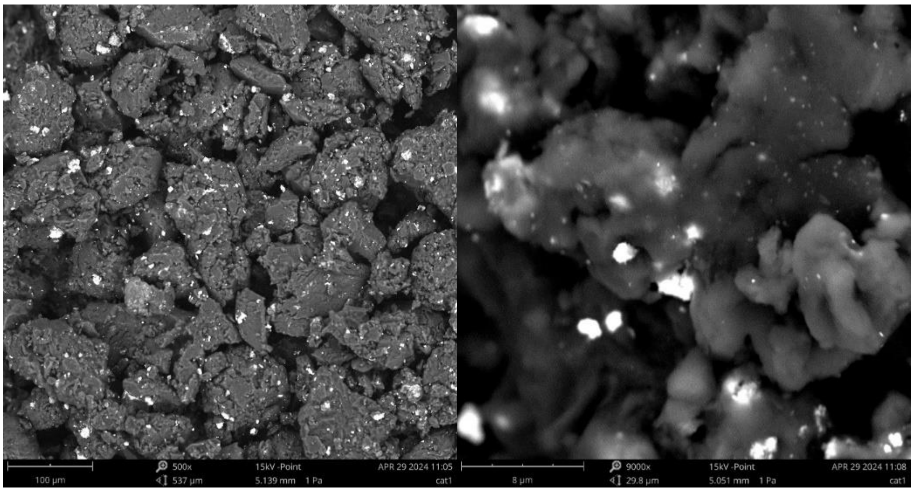


**Cat1**

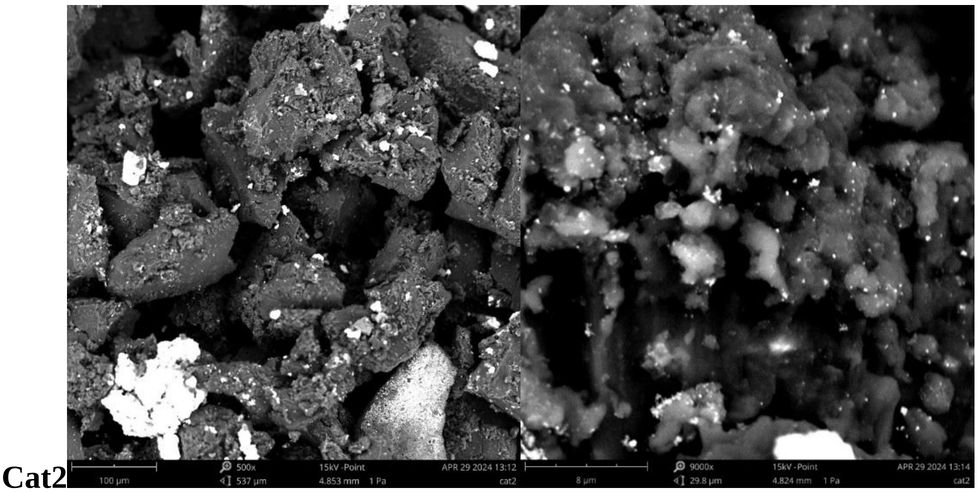


**Cat2**

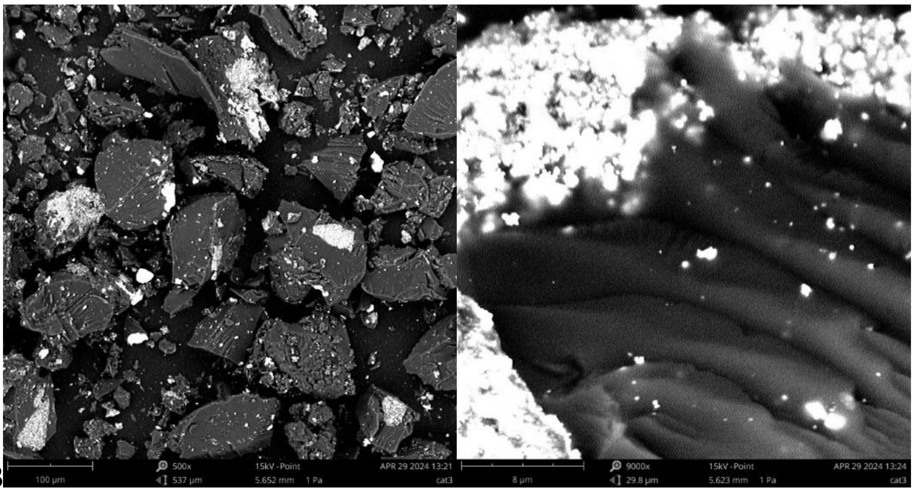

**Cat3**

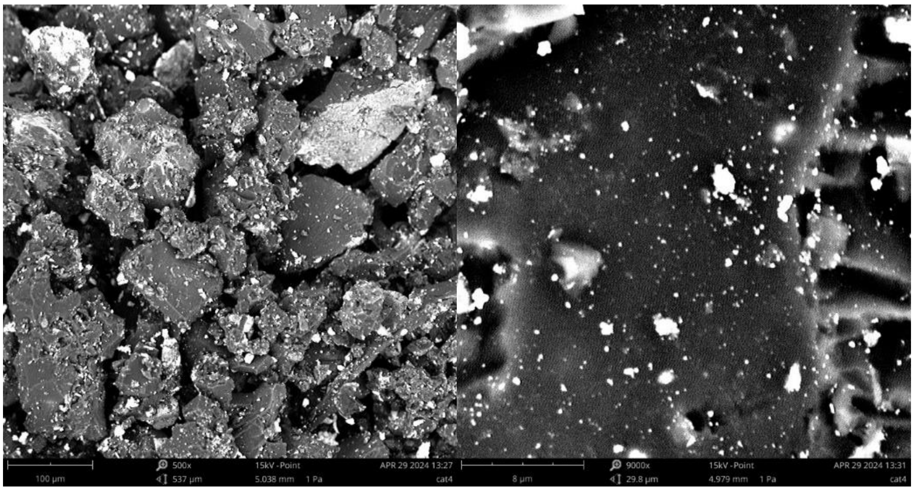

**Cat4**

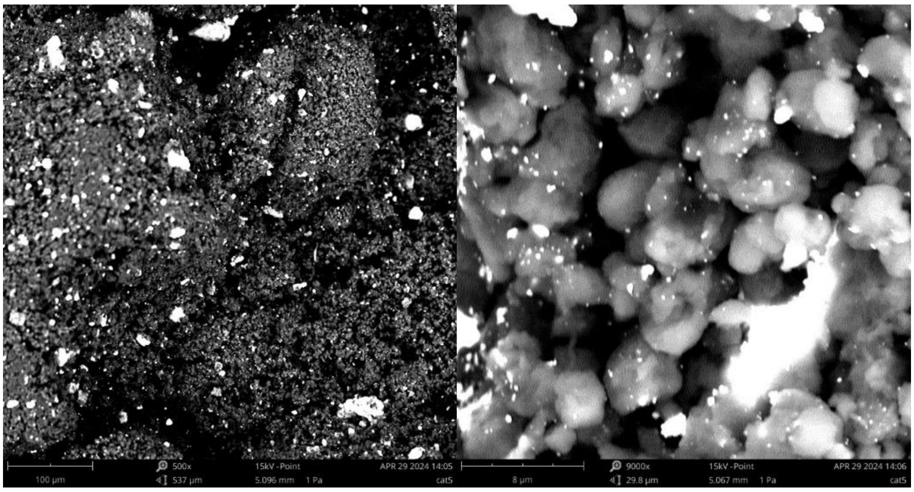

**Cat5**

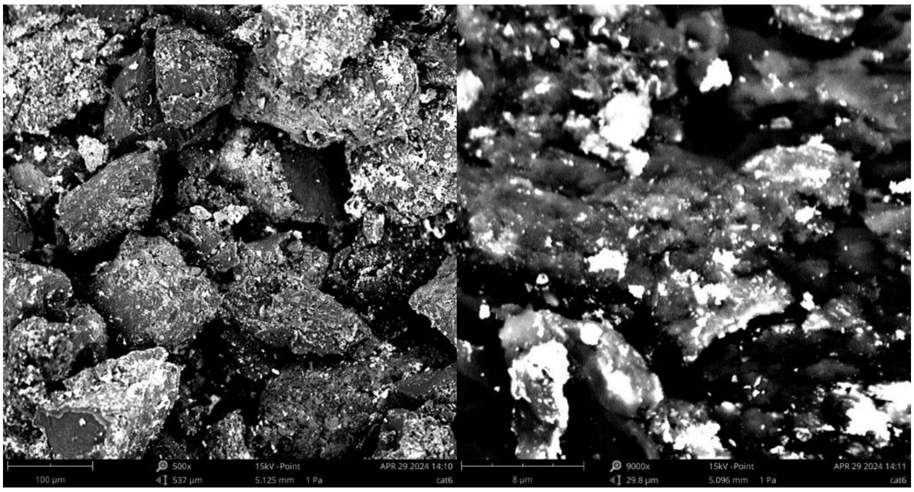

**Cat6**

**Figure S2. Histograms for photodegradation data.**

2.1 Percent photodegradation of dyes (data from Table 5)

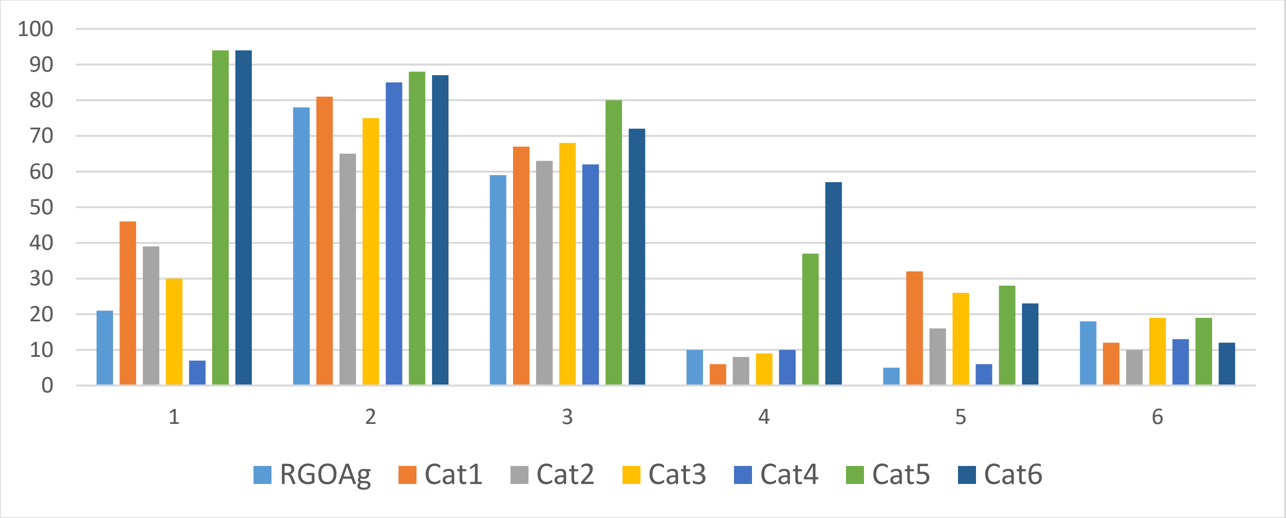


2.2 Percent photodegradation of drugs (data from Table 5)

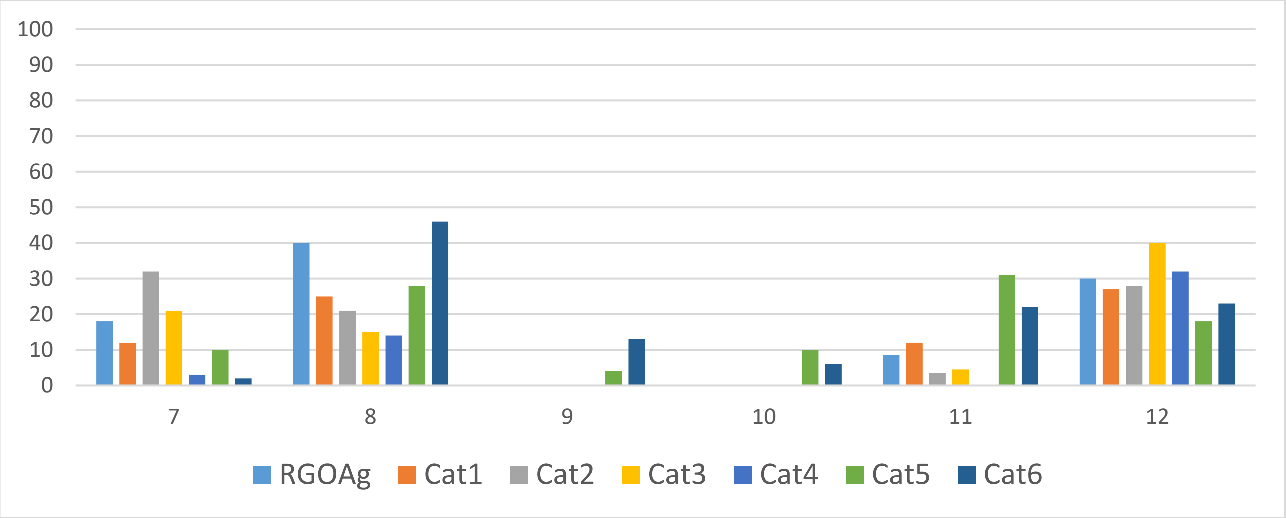


2.3 Photodegradation efficiency enhancements with respect to RGOAg (data from Table 7)

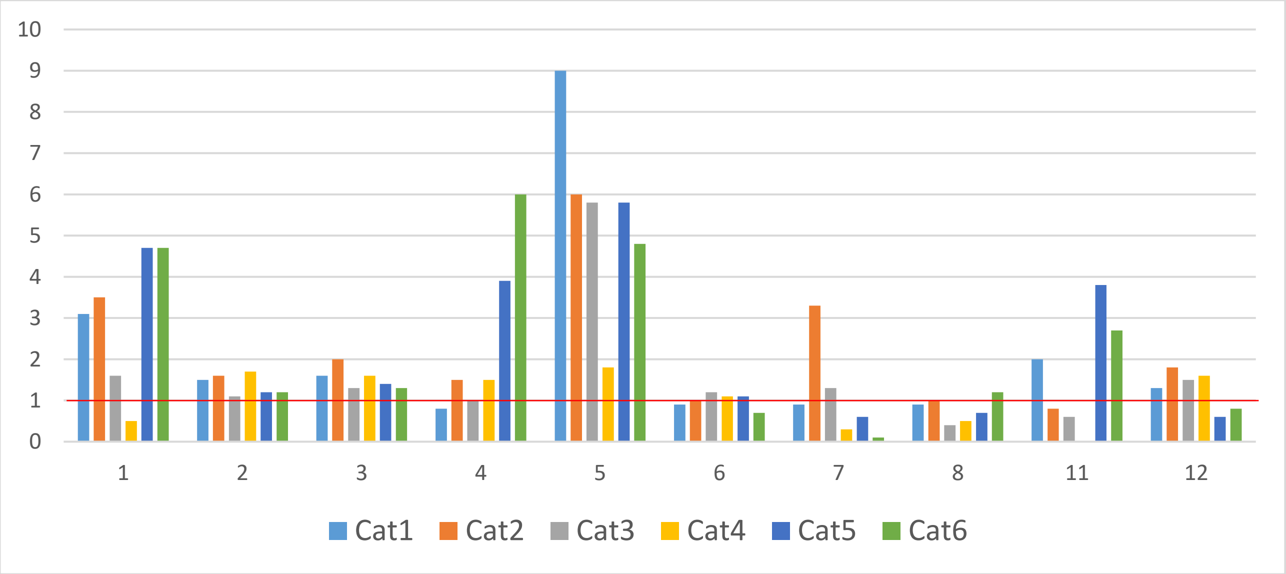

Supplement: Supplementary file 1 [file materials-17-02319-s001.zip › materials-2966338-supplementary.pdf]
